# Characteristics of left ventricular dysfunction in repaired tetralogy of Fallot: A multi-institutional deep learning analysis of regional strain and dyssynchrony

**DOI:** 10.1016/j.jocmr.2025.101886

**Published:** 2025-03-21

**Authors:** Brendan T. Crabb, Rahul S. Chandrupatla, Evan M. Masutani, Sophie Y. Wong, Sachin Govil, Silvia Montserrat, Susana Prat-González, Julián Vega-Adauy, Melany Atkins, Daniel Lorenzatti, Chiara Zocchi, Elena Panaioli, Nathalie Boddaert, Laith Alshawabkeh, Lewis Hahn, Sanjeet Hegde, Andrew D. McCulloch, Francesca Raimondi, Albert Hsiao

**Affiliations:** a University of California San Diego, La Jolla, California, USA; b Hospital Clínic Barcelona, Barcelona, Spain; c Chilean Institute of Cardiac Imaging, Santiago, Chile; d Inova Fairfax Hospital, Fairfax, Virginia, USA; e San Donato Hospital, Arezzo, Italy; f Rady Children’s Hospital San Diego, San Diego, California, USA; g Necker Enfants-Malades Hospital, Université de Paris, Paris, France

**Keywords:** Cardiovascular magnetic resonance (CMR), Deep learning, Myocardial strain, Congenital heart disease, Tetralogy of Fallot

## Abstract

**Background:**

Patients with repaired tetralogy of Fallot (rTOF) are commonly followed with cardiovascular magnetic resonance (CMR) imaging and frequently develop right ventricular (RV) dysfunction, which can be severe enough to impact left ventricular (LV) function in some patients. In this study, we sought to characterize patterns of LV dysfunction in this patient population using deep learning synthetic strain (DLSS), a fully automated deep learning algorithm capable of measuring regional LV strain and dyssynchrony.

**Methods:**

We retrospectively collected cine steady-state free precession (SSFP) MRI images from a multi-institutional cohort of 198 patients with rTOF and 21 healthy controls. Using DLSS, we measured LV strain and strain rate across 16 American Heart Association segments from short-axis cine SSFP images and compared these values to controls. We then performed a clustering analysis to identify unique patterns of LV contraction, using segmental peak strain and several measures of dyssynchrony. We further characterized these patterns by assessing their relationship to traditional MRI metrics of volume and function. Lastly, we assessed their impact on subsequent progression to pulmonary valve replacement (PVR) through a multivariate analysis.

**Results:**

Overall, patients with rTOF had decreased septal radial strain, increased lateral wall radial strain, and increased dyssynchrony relative to healthy controls. Clustering of rTOF patients identified four unique patterns of LV contraction. Most notably, patients in cluster 1 (n = 39) demonstrated an LV contraction pattern with paradoxical septal wall motion and severely reduced septal strain. These patients had significantly elevated RV end-diastolic volume relative to clusters 3 and 4 (153 ± 34 vs 127 ± 34 and 126 ± 31 mL/m^2^, analysis of variance *p* < 0.01). In the multivariate analysis, this contraction pattern was the only LV metric associated with future progression to PVR (heart rate = 2.69, *p* < 0.005). A smaller subset of patients (cluster 2, n = 29) showed reduced septal strain and LV ejection fraction despite synchronous ventricular contraction.

**Conclusion:**

Patients with rTOF demonstrate four unique patterns of LV dysfunction. Most commonly, but not exclusively, LV dysfunction is characterized by septal wall motion abnormalities and severely reduced septal strain. Patients with this pattern of LV dysfunction had concomitant RV dysfunction and rapid progression to PVR.

## Graphical abstract

**Figure:**
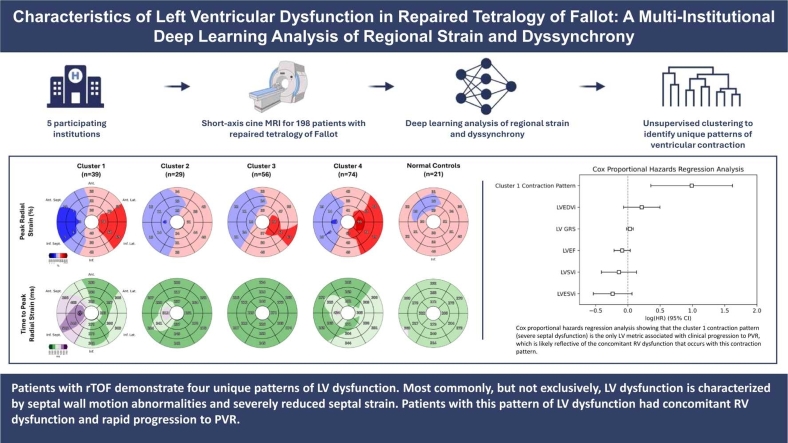

## 1 Background

Patients with repaired tetralogy of Fallot (rTOF), the most common cyanotic congenital heart defect, frequently develop progressive pulmonary regurgitation and right ventricular (RV) dilation following surgical repair of the RV outflow tract [1], [2], [3], [4], [5]. In some patients, RV dilation can be severe enough to impair left ventricular (LV) function, which has been independently associated with ventricular tachyarrhythmias, reduced exercise capacity, and sudden cardiac death [6], [7], [8], [9], [10], [11]. Consequently, patients are followed with cardiovascular magnetic resonance (CMR) imaging, the gold standard for evaluating biventricular size and function [12], [13].

Despite its impact on outcomes and clinical management, LV dysfunction in rTOF is still poorly understood [6], [14]. Recent studies have highlighted the presence of paradoxical interventricular septal wall motion, which suggests that ventricular-ventricular interactions are contributing [15], [16], [17]. However, it is unclear if these septal wall motion abnormalities fully explain the development of LV systolic dysfunction in rTOF. Similarly, it is unclear if the dysfunction is localized to the interventricular septum or if the entire LV demonstrates adverse remodeling [14], [18], [19]. Importantly, these questions cannot be answered using traditional MRI global metrics of LV function, such as ejection fraction (EF) or global strain, since these approaches lack regional granularity.

Recently, our group demonstrated the feasibility of using a deep learning-based technique to address this issue and quantify regional myocardial function [20]. This technique, called deep learning synthetic strain (DLSS), infers myocardial velocity directly from steady-state free precession (SSFP) CMR images, enabling the fully automated quantification of regional strain and dyssynchrony without user dependence. Importantly, this approach enables the automated characterization of large patient cohorts—a task that is typically labor and time intensive. In this study, we sought to leverage this approach to characterize patterns of LV dysfunction in a multi-center, international cohort of patients with rTOF.

## 2 Methods

This retrospective cohort study complied with European Union general data protection regulations on retrospective studies (authorization number: MR004: 20200509173610) for the site in France. For the site in Spain, all participants gave informed consent. For sites in the United States, studies were performed with local Institutional Review Board approval and a waiver of informed consent. For healthy volunteers, we obtained written informed consent according to a separate institutional review board protocol for CMR examinations of the volunteers.

### 2.1 Study population

We retrospectively identified clinical CMR examinations for 204 patients with rTOF from five medical centers, performed between January 2009 and March 2021. Participating institutions included the UC San Diego Health (n = 34), Rady Children’s Hospital (n = 81), Necker-Enfants Malades Hospital (n = 39), Inova Fairfax Hospital (n = 30), and the Hospital Clínic de Barcelona (n = 20). Eligible participants included pediatric and adult patients with TOF and available clinical contrast-enhanced CMR examinations. Patients with pulmonary atresia and multiple aortopulmonary collaterals were not excluded from this study. Similarly, patients with prior palliative Blalock-Taussig (BT) shunts were not excluded. None of the participants had pacemakers. Patients with non-diagnostic cardiac MR images (n = 2) were excluded.

### 2.2 Imaging protocol and image analysis

**Fig. 1:**
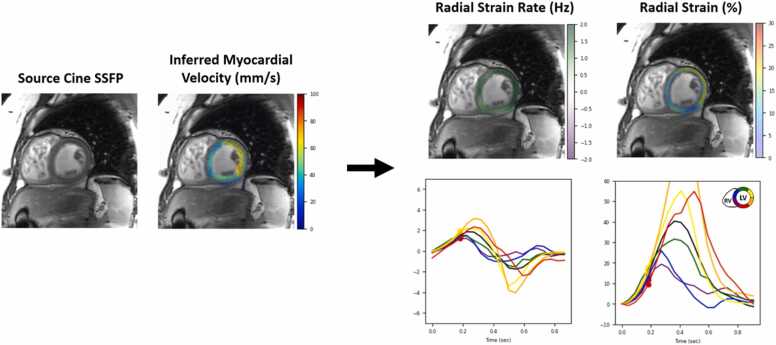
Deep learning synthetic strain analysis for a patient with tetralogy of Fallot, showing radial strain and strain rate maps and time curves derived from the inferred myocardial velocity. Using the strain and strain rate time curves, we calculate measures of dyssynchrony such as time to peak, strain-rate cross correlation, and uniformity ratio estimates. *SSFP* steady-state free precession.

Of the 204 studies included in the analysis, 153 were performed on a 1.5T or 3.0T GE Medical Systems magnetic resonance imaging (MRI) scanner (GE Healthcare, Chicago, Illinois, United States of America) and 51 were performed on a 1.5T Philips Medical Systems MRI scanner (Philips Healthcare, Best, The Netherlands). Specific CMR acquisition details for each institution are provided in Supplemental Table 1. For each patient, we retrospectively collected the short-axis cine SSFP series. Next, we manually identified an apical, mid ventricular, and basal slice for each patient. We then analyzed these slices using DLSS to measure strain, strain rate, and dyssynchrony. DLSS estimates strain and strain rate by inferring pixel-wise myocardial velocities directly from cine SSFP images (Fig. 1, Supplemental Video 1). These measurements are computationally decomposed into circumferential and radial components and subdivided into 16 segments according to the American Heart Association (AHA) model, with the apex region 17 excluded.

Supplementary material related to this article can be found online at doi:10.1016/j.jocmr.2025.101886.

The following is the Supplementary material related to this article Video S1..

Video S1 Deep learning synthetic strain analysis for a patient with TOF, showing strain maps and time curve plots derived from the inferred myocardial velocities

### 2.3 Calculation of biomechanical metrics

From DLSS measurements of regional strain, a variety of metrics to describe contraction strength and timing can be calculated. To assess contraction strength, we computed regional peak radial strain and radial strain rate. To assess contraction timing and dyssynchrony, we computed standard deviation of time to peak (sdTTP), maximum time to peak (TTP), strain-rate cross correlation (SRCC), and radial uniformity ratio estimates (RURE). Dyssynchrony metrics were computed separately for each slice. In this study, radial measures were preferentially used since septal wall motion abnormalities in rTOF occur primarily in the radial direction. Further details are provided in the Supplemental Material. We report reference ranges for each of these metrics and provide AHA bulls-eye plots demonstrating regional patterns of strain and dyssynchrony.

### 2.4 Correlation analysis

To assess the relationship between LV and RV function, as well as the relationship between DLSS metrics and traditional biometric variables, we performed a correlation analysis. Biometric variables included patient age, height, weight, body surface area (BSA), age at TOF surgical repair, prior pulmonary valve replacement (PVR) status, and time since PVR. We also included global measures of the RV and LV, including BSA indexed end-diastolic volume (EDVi), end-systolic volume (ESVi), stroke volume (SVi), and EF. Lastly, we added flow measurements including pulmonary net flow and pulmonary regurgitant fraction (PRF).

### 2.5 Hierarchical clustering analysis

To identify unique patterns of myocardial contraction, patients were clustered into groups using agglomerative hierarchical clustering. We standardized each metric to a mean of zero and standard deviation of one to weigh each metric equally. Importantly, the DLSS measures of regional strain and dyssynchrony were used directly in the clustering analysis, without dimensionality reduction, to maintain clinical explainability. For clustering, we utilized Ward’s method with a Euclidean distance metric. We selected the optimal number of clusters by using a majority rule of 23 indices of clustering quality using NbClust version 3.0.1 implemented in R version 4.4.2 (R Foundation for Statistical Computing, Vienna, Austria). We then assessed for differences between the discovered contraction patterns using the statistical analysis described below.

### 2.6 Longitudinal analysis

After completion of the clustering analysis, we collected longitudinal data regarding subsequent progression to PVR following CMR examination for each patient in the analysis. Data were collected through a retrospective review of the electronic health record system at each participating institution. Time zero was defined as the date of CMR examination in the original clustering analysis. Progression was defined as receiving a PVR. All patients were included in this analysis, including those who had previously undergone PVR, since these patients are monitored for and frequently require subsequent re-operations. We computed progression-free survival over time using Kaplan-Meier estimates with right-censored observations. We plotted survival curves for each cluster and assessed for differences in progression-free survival using pairwise log-rank tests. Furthermore, we conducted a Cox proportional hazards regression analysis to investigate the association between LV contraction patterns and progression to PVR when controlling for differences in LV volume and function between patients. We used a non-parametric Cox proportional hazards model using Breslow’s method. The included covariate factors were the contraction pattern, LV EF, LV global radial strain (gRS), and LV volume. For the survival analysis, we used the Python library Lifelines (lifelines v0.27.0, survival analysis in Python).

We also completed a sensitivity analysis, in which patients with a prior PVR were excluded from the survival analysis. Identical to the process described above, we computed progression-free survival over time using Kaplan-Meier estimates with right-censored observations for this new cohort. We plotted survival curves for each cluster and assessed for differences in progression-free survival using pairwise log-rank tests. This sensitivity analysis is reported in the Supplemental Material.

### 2.7 Statistical analysis

For the entire cohort, we report the mean and standard deviation for basic patient demographics, global LV and RV metrics, flow measurements, strain, and dyssynchrony metrics. Regional metrics of strain and dyssynchrony were compared to healthy controls using two-sided *t*-tests assuming equal population variances. The correlation between metrics was assessed using a Pearson correlation coefficient. Similarly, we report the mean and standard deviation of the above metrics for each cluster, or group of patients with similar contraction patterns, identified through the clustering analysis. We also report cardiopulmonary exercise testing (CPET) data for each cluster. We included CPET data that were performed within 3 years of the date of CMR examination at two institutions (UC San Diego Health and Rady Children’s Hospital). We assessed for statistically significant differences between all clusters using a one-way analysis of variance (ANOVA) and for pairwise statistical differences using a two-sided *t*-test assuming equal population variances. For categorical demographic information, we utilized a Pearson’s chi-square test to assess statistical significance. To assess differences in progression-free survival, we utilized pairwise log-rank tests. For all statistical tests, we set a type I error threshold of 0.01 (*p* < 0.01) to account for multiple comparisons based on the Bonferroni correction. Unless otherwise indicated, we used SciPy version 1.8.0, implemented in Python version 3.8.6 (Python Software Foundation, Wilmington, Delaware, USA) for all clustering and statistical analysis.

## 3 Results

### 3.1 Study population

Of the 204 patients, 6 (2.9%) had LV segmentation errors and were excluded before strain quantification. These infrequent errors, which precluded analysis with DLSS, were caused by poor image quality and the presence of motion artifacts. The remaining 198 patients included in the analysis had a mean age of 23.6 ± 13.0 years at the time of CMR examination. The mean age of initial surgical repair of the RV outflow tract was 2.8 ± 4.0 years. Thirty seven (18.7%, 37/198) patients had received PVR before the MRI examination with a mean time since PVR of 6.9 ± 6.5 years. One hundred and thirty-six (69%, 136/198) patients showed RV enlargement with RVEDVi ≥100 mL/m^2^ and 100 (51%) patients had pulmonary regurgitation with regurgitant fraction ≥25%, for a mean RVEDVi of 132.0 ± 35.5 mL/m^2^ and regurgitant fraction of 31.8 ± 18.1%. The cohort had a mean RVEF of 49.7 ± 8.2% and an LVEF of 57.9 ± 7.2%. These patients were compared to 21 healthy volunteers, with a mean age of 29 ± 5 years at the time of MRI examination. We report additional patient demographics, surgical information, and ventricular function and flow data in Table 1.

**Table 1:** Ventricular function and flow metrics for each institution and the entire cohort.

	UC San Diego Health (n = 34)	Necker-Enfants Malades Hospital (n = 39)	Rady Children’s Hospital (n = 79)	Inova Fairfax Hospital (n = 27)	Hospital Clínic de Barcelona (n = 19)	All patients (n = 198)
*Demographics*						
Age (y)	37.9±12.2	15.3±4.2	17.3±8.2	29.8±14.9	27.3±7.3	23.6±13.0
Weight (kg)	77.2±25.2	52.8±22.8	56.5±21.2	70.8±26.2	74.8±18.3	64.5±24.3
Height (cm)	167.1±10.9	154.9±24.0	155.8±19.8	162.0±12.7	172.8±10.9	160.9±18.1
BSA (m^2^)	1.8±0.3	1.5±0.3	1.5±0.4	1.8±0.3	1.9±0.2	1.6±0.4
Age at Repair (y)	2.9±2.2	0.4±0.3	3.9±5.8	2.5±3.0	4.6±2.4	2.8±4.0
Prior PVR (%)	15 (44%)	0 (0%)	11 (14%)	5 (18.5%)	6 (32%)	37 (19%)
Time since PVR (y)	6.4±6.5	-	7.2±3.8	13.2±9.2	2.7±2.1	6.9±6.5
	
*Volume and function*						
RVEDVi (mL/m^2^)	126.0±39.1	127.9±40.2	140.0±28.5	125.7±45.0	121.7±32.5	132.0±35.6
RVESVi (mL/m^2^)	68.3±26.5	61.7±22.9	67.5±17.5	70.5±34.0	68.8±23.6	66.6±22.3
RVSVi (mL/m^2^)	54.9±17.8	59.2±17.8	73.3±15.6	56.7±14.7	52.9±14.0	63.8±18.6
LVEDVi (mL/m^2^)	84.6±25.1	70.4±12.3	83.6±13.3	76.3±10.1	82.2±20.0	80.5±17.8
LVESVi (mL/m^2^)	39.1±19.1	29.6±8.0	33.8±7.8	34.2±9.3	40.9±12.4	34.7±12.2
LVSVi (mL/m^2^)	45.6±11.0	40.9±8.0	50.0±7.5	39.8±7.6	43.2±10.5	46.1±9.5
RVEF (%)	45.2±8.6	51.8±9.2	52.3±5.8	46.2±4.8	43.8±7.5	49.7±8.2
LVEF (%)	55.1±8.7	59.3±5.8	60.0±5.3	55.8±7.0	51.5±8.0	57.9±7.2
Peak VO_2_ (mL/kg/min)	23.4±5.3	-	29.6±9.0	-	-	26.9±8.2
Peak VO_2_ (% Predicted)	70.2±20.0	-	63.4±16.7	-	-	66.4±18.5
	
*Flow and regurgitation*						
Pulmonary net flow (L/min)	5.4±2.5	4.8±1.3	4.7±1.3	5.2±1.5	5.5±1.4	5.1±1.7
Pulmonary regurgitant fraction (%)	27.0±19.3	34.3±15.2	34.7±18.7	29.0±13.9	21.0±17.6	31.5±17.3

Demographic, ventricular function, cardiopulmonary exercise testing, and flow metrics for each institution and the entire cohort. Data is reported as means ± standard deviation.

*BSA* body surface area, *PVR* pulmonary valve replacement, *LV* left ventricle, *RV* right ventricle, *EDVi* end-diastolic volume, *ESVi* end-systolic volume, *SVi* stroke volume, *EF* ejection fraction, *VO_2_* volume of oxygen.

### 3.2 Regional mechanics for patients and controls

**Fig. 2:**
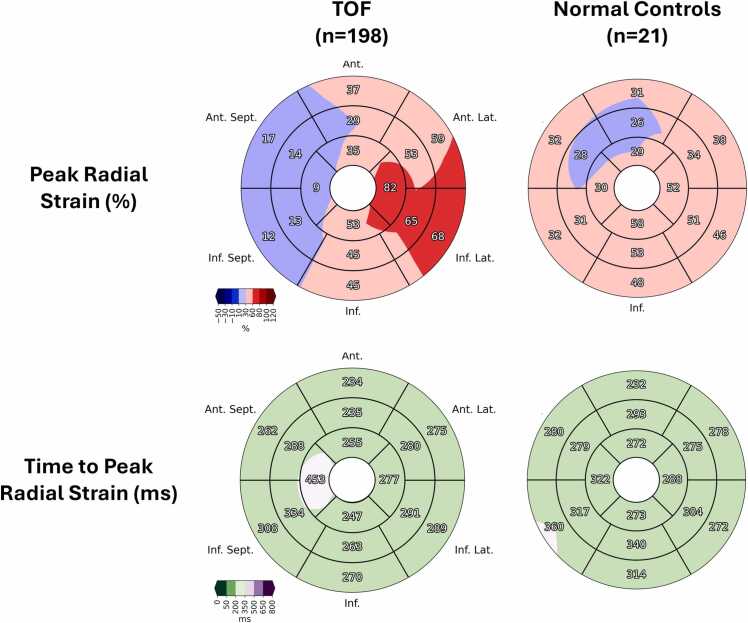
Sixteen-segment AHA bulls-eye plots of the mean peak radial strain and mean time to peak radial strain for patients with tetralogy of Fallot (n = 198) and healthy controls (n = 21). Relative to controls, patients with tetralogy of Fallot demonstrate reduced septal strain with compensation in the lateral wall. *AHA* American Heart Association, *TOF* tetralogy of Fallot.

A complete list of regional metrics of strain and dyssynchrony for rTOF patients and healthy controls is reported in Table 2. Moreover, AHA bulls-eye plots of the mean peak radial strain and mean TTP radial strain for rTOF patients and healthy controls are shown in Fig. 2. The patients with rTOF (n = 198) demonstrated significantly reduced radial strain in multiple segments relative to healthy controls. Most notably, they demonstrated reduced strain in the apical septal segment (10 ± 12% vs 31 ± 13%; *t*-test *p* < 0.001), midventricular inferoseptal and anteroseptal segments (14 ± 11% vs 32 ± 10%; *t*-test *p* < 0.001 and 15 ± 11% vs 28 ± 9%; *t*-test *p* < 0.001), and basal inferoseptal and anteroseptal segments (13 ± 10% vs 33 ± 10%; *t*-test *p* < 0.001 and 18 ± 13% vs 33 ± 7%; *t*-test *p* < 0.001). Interestingly, patients with rTOF demonstrate compensation in the lateral wall, with significantly increased radial strain relative to healthy controls in the apical lateral segment (83 ± 30% vs 52 ± 13%; *t*-test *p* < 0.001), midventricular inferolateral and anterolateral segments (65 ± 20% vs 51 ± 11%; *t*-test *p* < 0.01 and 54 ± 18% vs 35 ± 8%; *t*-test *p* < 0.001), and basal inferolateral and anterolateral segments (68 ± 23% vs 46 ± 9%; *t*-test *p* < 0.001 and 60 ± 26% vs 39 ± 7%; *t*-test *p* < 0.001). Patients with rTOF also demonstrated increased mechanical dyssynchrony relative to healthy controls in the apical segments (RURE 0.76 ± 0.10 vs 0.90 ± 0.05; *t*-test *p* < 0.001), midventricular segments (RURE 0.79 ± 0.10 vs 0.93 ± 0.04; *t*-test *p* < 0.001), and basal segments (RURE 0.82 ± 0.10 vs 0.95 ± 0.02; *t*-test *p* < 0.001).

**Table 2:** Regional metrics of strain and dyssynchrony for tetralogy of Fallot patients and normal controls.

Slice	Metric	TOF (n = 198)	Normal (n = 21)	*p-*value
Apical				
	Septal RS (%)	9.6±12.1	30.8±12.8	**<0.001**
	Anterior RS (%)	35.9±14.1	30.0±13.9	0.07
	Lateral RS (%)	82.6±30.0	52.4±13.1	**<0.001**
	Inferior RS (%)	53.8±23.2	58.8±14.8	0.33
	Global RS (%)	37.7±10.4	41.9±8.2	0.08
	Standard deviation TTP (ms)	131.6±78.4	60.8±65.9	**<0.001**
	Strain-rate cross correlation (ms)	282.0±179.3	72.9±37.1	**<0.001**
	RURE	0.76±0.10	0.90±0.05	**<0.001**
				
Midventricular				
	Inferoseptal RS (%)	13.7±11.4	31.5±9.7	**<0.001**
	Anteroseptal RS (%)	14.7±10.5	28.3±8.7	**<0.001**
	Anterior RS (%)	29.2±10.7	26.9±8.4	0.34
	Anterolateral RS (%)	54.1±17.8	34.6±8.3	**<0.001**
	Inferolateral RS (%)	65.3±20.3	51.4±11.3	**<0.01**
	Inferior RS (%)	45.2±17.6	53.1±9.9	0.04
	Global RS (%)	31.8±6.8	39.2±6.9	**<0.001**
	Standard deviation TTP (ms)	108.7±66.1	63.5±75.0	**<0.01**
	Strain-rate cross correlation (ms)	249.8±181.1	73.8±28.9	**<0.001**
	RURE	0.79±0.10	0.93±0.04	**<0.001**
				
Basal				
	Inferoseptal RS (%)	12.9±10.0	32.9±9.9	**<0.001**
	Anteroseptal RS (%)	18.0±12.5	32.7±6.9	**<0.001**
	Anterior RS (%)	37.8±15.1	31.4±7.4	0.05
	Anterolateral RS (%)	59.8±25.8	38.6±6.9	**<0.001**
	Inferolateral RS (%)	68.4±23.2	46.4±8.6	**<0.001**
	Inferior RS (%)	45.3±15.7	49.0±11.6	0.30
	Global RS (%)	34.0±7.0	38.7±5.4	**<0.01**
	Standard deviation TTP (ms)	99.0±63.1	69.2±56.6	0.04
	Strain-rate cross correlation (ms)	234.1±172.5	93.2±18.3	**<0.001**
	RURE	0.82±0.10	0.95±0.02	**<0.001**

Regional measures of radial strain and dyssynchrony for patients with repaired tetralogy of Fallot vs normal controls. *p values are calculated using a pairwise t-test. Significant values (p < 0.01) are bolded*.

*RS* radial strain, *TTP* time to peak, *RURE* radial uniformity ratio estimate, *TOF* tetralogy of Fallot.

### 3.3 Correlation analysis

We performed multiple direct correlation analyses to assess the relationship between RV and LV metrics. Most notably, LVEF showed a modest positive correlation with RVEF (*r* = 0.57, *p* < 0.001) and a weak correlation with RVEDVi (*r* = −0.20, *p* < 0.05). For the regional metrics of strain and dyssynchrony, radial strain showed a modest positive correlation with both LVEF and RVEF and a weak to modest negative correlation with LV and RV volumes (*r* = 0.23 to 0.51 for EFs, *r* = −0.07 to –0.28 for ventricular volumes). Measures of LV dyssynchrony, including sdTTP, SRCC, and RURE, each modestly correlated with pulmonary valve regurgitant fraction (*r* = 0.17 to 0.35).

### 3.4 Clustering analysis

**Fig. 3:**
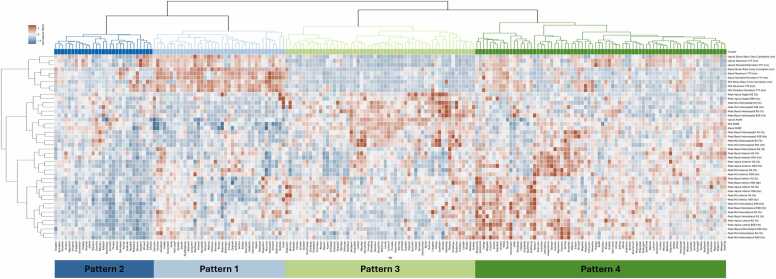
Hierarchical clustering of tetralogy of Fallot patients into unique contraction patterns using standardized regional strain and dyssynchrony measurements. Patient phonetic IDs are shown on the x-axis with color codes used to distinguish each phenotypic cluster. For each metric, the data are normalized and colored such that values greater than the mean are red, while values less than the mean are blue. We discovered multiple distinct contraction patterns from the regional measures of LV contraction strength and timing.

**Fig. 4:**
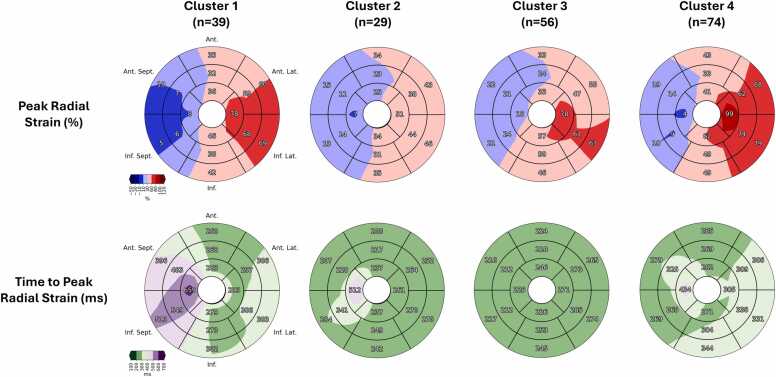
Sixteen-segment AHA bulls-eye plots of the mean peak radial strain and mean time to peak radial strain for clusters 1, 2, 3, and 4. Clusters 1, 3, and 4 demonstrate decreased strains in the septal segments, with compensation in the lateral wall. In contrast, cluster 2 demonstrates globally reduced radial strains without compensation in the lateral wall. Cluster 1 was also characterized by significantly increased time to peak radial strain (indicates increased dyssynchrony) in the apical, mid, and basal septal segments. *AHA* American Heart Association.

**Fig. 5:**
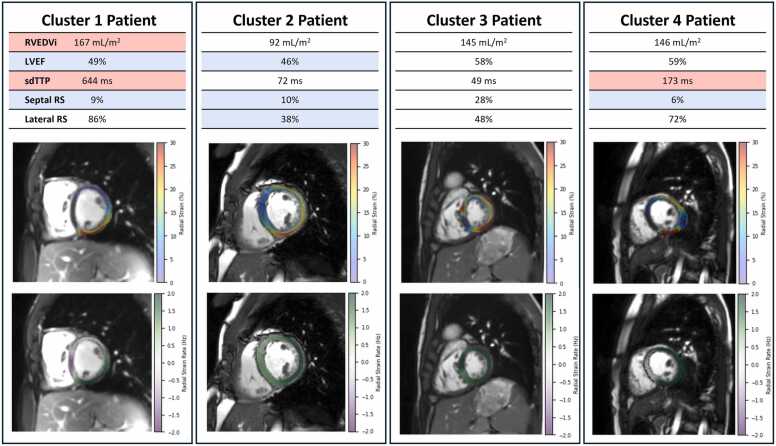
Representative patients from clusters 1, 2, 3, and 4. The cluster 1 patient demonstrates evidence of RV volume overload (increased RVEDVi) and septal dysfunction (increased dyssynchrony, decreased septal radial strain). Representative of cluster 2 is a patient with decreased LV function without evidence of RV volume overload or dyssynchrony. Representative of cluster 3 is a patient with adequate compensation in both the right and left ventricles. Representative of cluster 4 is a patient with mild apical dyssynchrony and septal dysfunction. *LV* left ventricle, *RV* right ventricle, *EDVi* end-diastolic volume, *EF* ejection fraction, *RS* radial strain, *sdTTP* standard deviation of time to peak.

The 198 patients with rTOF were clustered by radial strain and dyssynchrony metrics to identify groups of patients with similar regional patterns of LV contraction. This clustering analysis identified four distinct patterns of LV contraction within this patient population. The resultant dendrogram and heatmap of standardized metrics from this clustering analysis are shown in Fig. 3. To better visualize the mechanical differences between each contraction pattern, we provide bull’s-eye plots of the mean peak radial strain value and radial strain TTP (Fig. 4) for each AHA segment in each cluster. Representative patient examples for clusters 1, 2, 3, and 4 are shown in Fig. 5 and Supplemental Video 2.

Supplementary material related to this article can be found online at doi:10.1016/j.jocmr.2025.101886.

The following is the Supplementary material related to this article Video S2..

Video S2 Deep learning synthetic strain analysis for representative patients from clusters 1, 2, 3, and 4

**Fig. 6:**
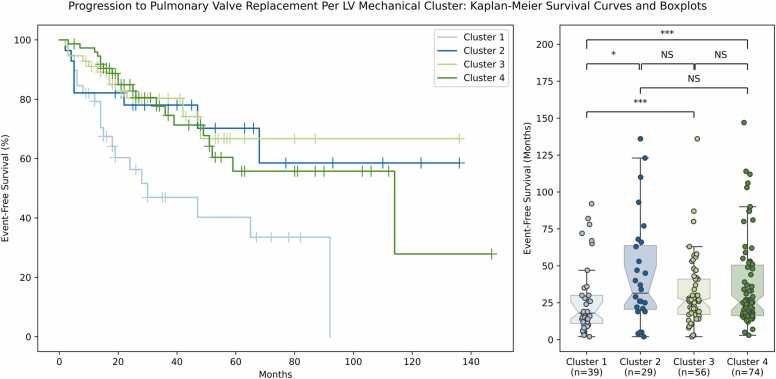
Kaplan-Meier survival curves and boxplots showing time since CMR evaluation until progression to PVR for patients in LV mechanical clusters 1, 2, 3, and 4. Patients with the cluster 1 contraction pattern, which was characterized by dyssynchrony and decreased radial strain in the anteroseptal and inferoseptal segments, showed a statistically significant decrease in survival relative to the well-compensated patients in clusters 3 and 4. Significance was assessed with pairwise log-rank tests. *p < 0.05; ***p < 0.001; *NS* not statistically significant, *LV* left ventricular, *CMR* cardiovascular magnetic resonance, *PVR* pulmonary valve replacement.

The cluster 1 contraction pattern (n = 39 patients) was characterized by decreased radial strain in mid anteroseptal, mid inferoseptal, basal anteroseptal, and basal inferoseptal segments relative to patients in clusters 2, 3, and 4 (*p* < 0.001). Importantly, the cluster 1 pattern was also characterized by increased dyssynchrony, with significantly greater sdTTP and SRCC and significantly lower RURE in the mid and basal slices relative to clusters 2, 3, and 4 (*p* < 0.001) (Fig. 6).

The cluster 2 contraction pattern (n = 29 patients) was characterized by globally diminished strains, particularly in lateral segments. Patients in cluster 2 had lower radial strain in mid anterolateral, mid inferolateral, and basal inferolateral segments than patients in clusters 1, 3, and 4 (*p* < 0.001). While differences in strain were most pronounced in the lateral segments, patients in cluster 2 also demonstrated lower radial strain than patients in cluster 3 in mid anteroseptal and basal anteroseptal segments. Importantly, patients in cluster 2 showed minimal evidence of dyssynchrony, with sdTTP, SRCC, and RURE values that were not statistically different than cluster 4 and only mildly increased relative to cluster 3.

The cluster 3 contraction pattern (n = 56 patients) was characterized by the highest radial strain in multiple segments, including the mid anteroseptal, mid inferoseptal, basal anteroseptal, and basal inferoseptal segments (*p* < 0.001). Further, these patients did not demonstrate any evidence of dyssynchrony—patients in this cohort had the lowest sdTTP and SRCC and highest RURE in the apical, mid, and basal slices of all four clusters.

The cluster 4 contraction pattern (n = 74) was demonstrated by the largest proportion of patients in our cohort and was characterized by preserved radial strain, particularly in the lateral segments. Notably, this group had the highest gRS in the apical, mid ventricular, and basal slices (*p* < 0.001). Despite having adequate strain, this group demonstrated evidence of mild dyssynchrony, with increased sdTTP and SRCC and lower RURE than patients in clusters 1 and 3 in the apical, mid, and basal slices.

We provide tables of the regional and gRS and radial strain rate for clusters 1, 2, 3, 4, and the entire cohort in Supplemental Table 2. Moreover, we report all dyssynchrony metrics for clusters 1, 2, 3, and 4 in Supplemental Table 3.

### 3.5 Relationship of clusters with ventricular function and flow

We further characterized each cluster by tabulating patient demographic information, ventricular volume and function, CPET, and flow and regurgitation data for each cluster in Table 3. Patients in cluster 1 (reduced septal strain and increased dyssynchrony) showed RV enlargement with significantly higher RVEDVi (153.0 ± 33.9 vs 127.0 ± 33.7 and 125.6 ± 31.2 mL/m^2^ for clusters 3 and 4; ANOVA *p* < 0.01) and PRF (40.7 ± 13.3% vs 23.3 ± 16.7% and 20.7 ± 16.9% for clusters 2 and 3; ANOVA *p* < 0.001). Thirty-two (82%) patients had dilated right ventricles (RVEDVi ≥100 mL/m^2^) and 25 (64%) had substantial pulmonary regurgitation with regurgitant fraction ≥25%. Only 2 patients in cluster 1 (5.1%) had PVR before the MRI examination used for this analysis, compared with 9 (31.0%), 16 (29%), and 10 (14%) patients in clusters 2, 3, and 4, respectively.

**Table 3:** Ventricular function and flow metrics for patients with each pattern of LV contraction (clusters).

	Cluster 1 (n = 39)	Cluster 2 (n = 29)	Cluster 3 (n = 56)	Cluster 4 (n = 74)	*p*-value
*Demographics*					
Age (y)	22.3±10.7	30.7±13.0	24.0±13.9	21.4±12.5	0.02
Weight (kg)	67.3±33.6	70.3±18.6	102.5±50.5	109.8±49.5	0.69
Height (cm)	157.4±22.1	169.4±17.6	129.1±49.3	104.5±55.8	0.03
BSA (m^2^)	1.7±0.4	1.8±0.3	1.7±0.4	1.5±0.3	0.01
Age at repair (y)	3.4±4.1	4.4±5.0	2.4±3.4	2.0±3.7	0.05
Prior PVR (%)	2 (5.1%)^a^	9 (31.0%)	16 (29%)^b^	10 (14%)	**<0.01**
Time since PVR (y)	4.5±4.5	5.1±5.6	6.9±6.0	9.2±7.6	0.56
					
*Volume and function*					
RVEDVi (mL/m^2^)	153.0±33.9^a^^,^^c^	130.5±37.4	127.0±33.7^b^	125.6±31.2^b^	**<0.01**
RVESVi (mL/m^2^)	73.2±19.1	71.8±26.5	66.7±21.5	60.9±19.4	0.04
RVSVi (mL/m^2^)	73.3±15.9^a^^,^^d^	58.8±15.9^b^	58.5±17.7^b^	65.7±19.3	**<0.01**
LVEDVi (mL/m^2^)	83.3±14.3	92.1±28.4	57.7±29.1	57.7±28.3	0.10
LVESVi (mL/m^2^)	37.1±8.0	45.6±22.3^c^	38.6±8.8	36.2±8.8^d^	**<0.01**
LVSVi (mL/m^2^)	46.3±9.8	48.0±11.5	44.6±14.1	45.5±9.6	0.05
RVEF (%)	49.3±6.2^c^	45.7±7.2^c^	47.4±9.4^c^	53.7±7.0^a^^,^^b^^,^^d^	**<0.001**
LVEF (%)	56.0±5.6^c^	52.6±10.2^a^^,^^c^	58.1±5.6^c^,	61.0±5.8^a^^,^^b^^,^^d^	**<0.001**
Peak VO_2_ (mL/kg/min)	27.4±8.5	27.1±8.1	25.3±7.8	27.7±9.1	0.86
Peak VO_2_ (% Predicted)	70.0±25.7	73.1±19.3	57.0±13.3	66.5±16.2	0.10
					
*Flow and regurgitation*					
Pulmonary net flow (L/min)	5.2±2.8	5.2±1.4	5.2±1.6	4.9±1.0	0.78
Pulmonary regurgitant fraction (%)	40.7±13.3^a^^,^^d^	23.3±16.7^b^^,^^c^	20.7±16.9^b^^,^^c^	39.1±16.3^a^^,^^d^	**<0.001**

Demographic, ventricular function, cardiopulmonary exercise testing, and flow metrics for patients with each pattern of LV contraction (clusters). Data are reported as the mean and standard deviation. *p values are calculated between clusters using a one-way ANOVA for continuous variables and a Pearson’s chi-square test for categorical variables. Significant values (p < 0.01) are bolded*.

*ANOVA* analysis of variance, *BSA* body surface area, *PVR* pulmonary valve replacement, *LV* left ventricle, *RV* right ventricle, *EDVi* end-diastolic volume, *ESVi* end-systolic volume, *SVi* stroke volume, *EF* ejection fraction, *VO*_2_ volume of oxygen.

a Post-hoc pairwise analysis: significantly different from 3

b Post-hoc pairwise analysis: significantly different from 1

c Post-hoc pairwise analysis: significantly different from 4

d Post-hoc pairwise analysis: significantly different from 2

Cluster 2 patients (globally reduced strain without dyssynchrony) showed normal RV volumes with a mean RVEDVi that was not statistically greater than patients in cluster 3 or 4. However, cluster 2 still had the lowest LVEF of all four clusters (52.6 ± 10.2% vs 56.0 ± 5.6%, 58.1 ± 5.6%, and 61.0 ± 5.8% for clusters 1, 3, and 4; ANOVA *p* < 0.001). Nevertheless, 14 (48%) patients had LVEF exceeding 55%. Patients in cluster 2 trended toward older (30.7 ± 13.0 vs 22.3 ± 10.7, 24.0 ± 13.9, and 21.4 ± 12.5 years for clusters 1, 3, and 4; ANOVA *p* = 0.02) and were more likely to have received a prior PVR (31% vs 5%, 29%, and 14% for clusters 1, 3, and 4; chi^2^ *p* < 0.01).

Patients in cluster 3 (preserved strain and absent dyssynchrony) had a lower mean RVEDVi than patients in cluster 1 (127.0 ± 33.7 vs 153.0 ± 33.9 mL/m^2^, *t*-test *p* < 0.01). Notably, patients with this contraction pattern had no significant differences in volume relative to the well-compensated patients in cluster 4. Patients in cluster 3 also demonstrated the lowest PRF of all clusters (20.7 ± 16.9% vs 40.7 ± 13.3%, 23.3 ± 16.7%, and 39.1 ± 16.3% for clusters 1, 2, and 4, ANOVA *p* < 0.001).

Patients in cluster 4 (preserved strain and mild dyssynchrony) had the lowest mean RVEDVi, RVESVi, LVEDVi, and LVESVi of all four clusters. Cluster 4 also had the highest LVEF (61.0 ± 5.8% vs 56.0 ± 5.6%, 52.6 ± 10.2%, and 58.1 ± 5.6% for clusters 1, 2, and 3; ANOVA *p* < 0.001) and the highest RVEF of all four clusters (53.7 ± 7.0% vs 49.3 ± 6.2%, 45.7 ± 7.2%, and 47.4 ± 9.4% for clusters 1, 2, and 3; ANOVA *p* < 0.001). Notably, patients with this contraction pattern still had pulmonary regurgitation, with a higher mean regurgitant fraction than patients in cluster 2 or 3 (39.1 ± 16.3% vs 20.7 ± 16.9% and 23.3 ± 16.7% for clusters 2 and 3; ANOVA *p* < 0.001).

### 3.6 Survival analysis: progression to pulmonary valve replacement

**Fig. 7:**
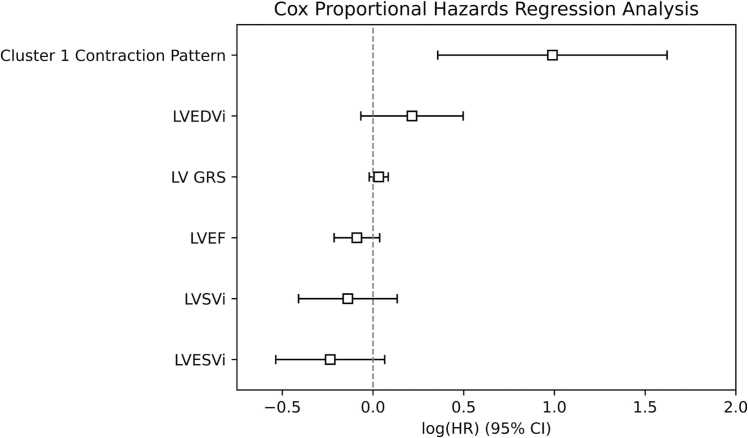
Cox proportional hazards regression analysis showing that the cluster 1 contraction pattern (severe septal dysfunction) is the only LV metric associated with clinical progression to PVR, which is likely reflective of the concomitant RV dysfunction that occurs with this contraction pattern. Of note, when controlling for this pattern of LV dysfunction, no other LV metric remains independently associated with progression to PVR. *LV* left ventricle, *RV* right ventricle, *PVR* pulmonary valve replacement, *EDVi* indexed end diastolic volume, *GRS* global radial strain, *EF* ejection fraction, *SVi* indexed stroke volume, *ESVi* indexed end-systolic volume.

We assessed subsequent progression to PVR in all 198 patients. The mean follow-up time was 4.8 ± 3.6 years (range 0.4–13.3 years). A total of 60 patients (30%, 60/198) received a PVR during the follow-up period, with a mean time to PVR of 34.1 ± 28.4 months (range 2–147 months) after the date of MRI examination in the original clustering analysis. Patients in cluster 1 exhibited more rapid progression to PVR than patients in clusters 2, 3, and 4 (26 ± 24 vs 43 ± 36, 31 ± 23, and 37 ± 30 months, log-rank *p* < 0.001). We show Kaplan-Meier survival curves for clusters 1, 2, 3, and 4, as well as boxplots of progression-free survival time for each cluster, in Fig. 5. Progression to PVR was also analyzed using a Cox proportional hazards regression analysis. Even when controlling for differences in LVEF, LV gRS, LVEDVi, LVESVi, and LVSVi between patients, the cluster 1 contraction pattern showed a statistically significant decrease in time to PVR (heart rate [HR] 2.69 (95% CI 1.43–5.06), *p* < 0.005). Of all the LV covariates included in the analysis, this pattern was the only factor that was independently and significantly associated with progression to PVR. Notably, LVEF was not found to be significantly associated with progression to PVR after controlling for other factors (HR 0.91 (95% CI 0.81–1.04), *p* = 0.16). A summary of the Cox proportional hazards regression analysis can be seen in Table 4 and Fig. 7.

**Table 4:** Cox proportional hazards regression analysis.

Covariate	Hazard ratio (95% CI)	*p*-value
Cluster 1 contraction pattern	2.69 (1.43–5.06)	<0.005
LVEDVi (mL/m^2^)	1.24 (0.94–1.64)	0.14
LV gRS (%)	1.03 (0.98–1.09)	0.24
LVEF (%)	0.91 (0.81–1.04)	0.16
LVSVi (mL/m^2^)	0.87 (0.66–1.14)	0.32
LVESVi (mL/m^2^)	0.79 (0.59–1.07)	0.12

Multivariate Cox proportional hazards regression analysis showing the association between LV covariates and progression to PVR. In this multivariate analysis, the cluster 1 contraction pattern (severe septal dysfunction) is the only LV metric associated with clinical progression to PVR, which is likely reflective of the concomitant RV dysfunction that occurs with this pattern of LV dysfunction. Data is reported as the hazard ratio and 95% confidence interval.

*LV* left ventricle, *RV* right ventricle, *EDVi* end-diastolic volume, *ESVi* end-systolic volume, *SVi* stroke volume, *EF* ejection fraction, *PVR* pulmonary valve replacement, *gRS* global radial strain.

## 4 Discussion

In this multi-center, international cohort of 198 patients with rTOF, we perform the largest analysis of LV regional strain and dyssynchrony in rTOF to date, facilitated by the application of a deep learning strain algorithm on historical MR images [21], [22], [23], [24], [25], [26]. In this cohort, LV dysfunction was strongly correlated with RV dysfunction and characterized by reduced strain in the interventricular septum and increased mechanical dyssynchrony relative to healthy controls. Moreover, an unsupervised clustering analysis of regional strain and dyssynchrony metrics identified four unique patterns of LV contraction. Most notably, a group of patients demonstrated LV dysfunction characterized by severely reduced septal strain and paradoxical septal wall motion, which interestingly has been observed in earlier, smaller studies using echocardiography and dedicated myocardial tagging [23], [24]. These patients had significant concomitant RV dysfunction and rapid progression to PVR. In fact, when controlling for this pattern of dysfunction through a multivariable analysis, LVEF was no longer predictive of future progression to PVR. These findings suggest that reduced septal strain and dyssynchrony may be more sensitive indicators of concomitant RV dysfunction and adverse ventricular-ventricular interactions than a reduced LVEF. Future studies are needed to investigate the incremental prognostic value of regional strain and dyssynchrony relative to LVEF for the prediction of adverse outcomes.

In contrast to prior studies, we also identified a group of patients (cluster 2) with reduced strain in both the interventricular septum and the lateral segments. Moreover, this LV dysfunction occurred even in the absence of septal wall motion abnormalities. Patients in this group tended to be older (30.7 ± 13.0 years) and were more likely to have had a prior PVR. Several patients in this group had prior palliative BT shunts and were of older age, which may have contributed to lower global LV strain. However, the frequency of BT shunts in this cluster was not statistically different from the other groups. It is also worth noting that 14 (48%, 14/29) patients in this group had a normal LVEF >55% and thus could not have been identified using traditional measures of LV function.

Taken together, these findings support the conclusion that adverse ventricular-ventricular interactions and paradoxical septal wall motion contribute to LV dysfunction in TOF (cluster 1). However, alternative mechanisms likely also contribute, since some patients demonstrate LV dysfunction even in the absence of these findings (cluster 2). Previous studies have proposed several potential alternative mechanisms for the development of LV dysfunction in rTOF, including chronic cyanosis due to palliative shunting before definitive surgical repair or intraoperative hypoxic injury at the time of surgical intervention [27], [28], which may be contributing.

From a clinical perspective, the distinct patterns of LV dysfunction discovered in this study raise several questions regarding the optimal clinical management of rTOF patients. Most notably, the patterns of LV dysfunction may impact future risk of adverse cardiac events or response to surgical intervention. Consequently, optimal clinical management might depend on both the degree and pattern of dysfunction displayed by the patient. In previous studies, not all patients experienced a clinically significant improvement in LVEF following PVR [29], [30]. It is possible that these different patterns of LV dysfunction, and their association with ventricular-ventricular interactions, could be used to predict which patients will have a clinically significant improvement in LVEF following PVR [29]. Future studies are warranted to determine if these patterns of LV dysfunction can be used to improve prognostication and optimize clinical management.

## 5 Limitations

This study was limited to the investigation of LV strain. We anticipate that extension of the algorithm to analyze regional RV strain will likely further improve characterization of this population. Similarly, we focused our analysis on radial strain, without investigating circumferential or longitudinal strain, which may be correlated and further stratify patient groups. Investigating longitudinal strain will require developing similar deep learning algorithms capable of operating on long-axis images. Nevertheless, with radial components alone, we identified distinct and clinically relevant contraction patterns. We limited our analysis to four primary subgroups to maintain statistical power; however, it is feasible that the clusters could be further subdivided to provide additional pathophysiological insights. DLSS offers the potential added advantage of regional measurements without user dependence, facilitating larger population studies due to its ability to automate analyses. However, we observed an algorithm failure rate of approximately 2.9% (n = 6 of 204 cases), where poor image quality and motion artifacts precluded an accurate LV myocardial segmentation.

## 6 Conclusions

Patients with rTOF demonstrate four unique patterns of LV dysfunction, including a group of patients with diminished septal strain and increased septal dyssynchrony. This pattern is highly correlated with RV dysfunction and future progression to PVR, suggesting ventricular-ventricular interactions are contributing. However, some patients demonstrate diminished LV strain even in the absence of septal dyssynchrony. These patterns were discovered using a fully automated deep learning analysis of regional LV mechanics, highlighting the potential for deep learning algorithms to phenotypically characterize large patient cohorts and provide new pathophysiological insights.

## Funding

The primary author was supported by the 10.13039/100013915Sarnoff Cardiovascular Research Foundation, an Award from the 10.13039/100000968American Heart Association and The Children’s Heart Foundation, and an 10.13039/100000002NIH T32 Fellowship (BTC). Several investigators were supported by the Congenital Heart Disease Cardiac Atlas Project, NIH grant R01HL121754 (A.D.M.). R.S.C. was supported by the Altman Clinical and Translational Research Institute MedGap program. Additional in-kind support was provided by Oracle for Research (A.H.) for cloud computation. The reported funding organizations had no role in the design of the study and collection, analysis, interpretation of data, or manuscript writing.

## Author contributions

**Nathalie Boddaert:** Data curation. **Rahul S. Chandrupatla:** Formal analysis, Data curation. **Laith Alshawabkeh:** Data curation, Conceptualization. **Evan M. Masutani:** Software. **Chiara Zocchi:** Data curation. **Elena Panaioli:** Data curation. **Brendan T. Crabb:** Writing – review & editing, Writing – original draft, Visualization, Validation, Software, Methodology, Investigation, Formal analysis, Data curation, Conceptualization. **Silvia Montserrat:** Data curation. **Andrew D. McCulloch:** Writing – review & editing, Supervision, Methodology, Conceptualization. **Susana Prat-Gonzalez:** Data curation. **Francesca Raimondi:** Writing – review & editing, Validation, Supervision, Methodology, Investigation, Data curation, Conceptualization. **Lewis Hahn:** Writing – review & editing, Data curation, Conceptualization. **Sophie Y. Wong:** Writing – review & editing, Data curation. **Sachin Govil:** Writing – review & editing, Conceptualization. **Sanjeet Hegde:** Writing – review & editing, Methodology, Data curation, Conceptualization. **Melany Atkins:** Data curation, Conceptualization. **Daniel Lorenzatti:** Validation, Data curation. **Albert Hsiao:** Writing – review & editing, Writing – original draft, Visualization, Validation, Supervision, Software, Resources, Project administration, Methodology, Investigation, Formal analysis, Data curation, Conceptualization. **Julian Vega-Adauy:** Data curation.

## Ethics approval and consent

Deidentified datasets employed in this study were contributed from 5 clinical centers (UC San Diego Health, Rady Children’s Hospital, Necker-Enfants Malades Hospital, Inova Fairfax Hospital, and the Hospital Clínic de Barcelona). This study complied with European Union general data protection regulations on retrospective studies (authorization number: MR004: 20200509173610) for the site in France. Moreover, for the site in Spain, all participants gave informed consent. For sites in the United States, studies were performed with local Institutional Review Board approval and a waiver of informed consent.

## Consent for publication

Not applicable.

## Declaration of competing interests

The authors declare the following financial interests/personal relationships which may be considered as potential competing interests: M.A. serves as a consultant for GE Healthcare. A.H. receives research grant support from GE Healthcare and was previously a co-founder with ownership interests in Arterys, which has been acquired by Tempus AI. A.D.M. is a co-founder with equity interest in Insilicomed and Vektor Medical. The other authors report no conflicts.
