# Supplementary material for: Characteristics of left ventricular dysfunction in repaired tetralogy of Fallot: A multi-institutional deep learning analysis of regional strain and dyssynchrony
Source: J Cardiovasc Magn Reson. 2025 Mar 21;27(1):101886. doi: 10.1016/j.jocmr.2025.101886 (PMC12182814; doi:10.1016/j.jocmr.2025.101886)
Supplement: Supplementary file 3 — Supplementary material [file mmc1.docx]

**Supplemental Material**

**Supplemental Methods**

**Calculation of Dyssynchrony Metrics**

To measure contraction timing and dyssynchrony, we employed time to peak (TTP) standard deviation, maximum TTP, strain-rate cross correlation and uniformity ratio estimates. We calculated measures of dyssynchrony in the radial direction using all relevant segments in the apical, mid ventricular, or basal slice.

***Time-to-Peak***

TTP was calculated as the time delay between end-diastole and the peak radial or circumferential strain for each of the 16 AHA segments. From these values, we calculate and report the standard deviation of segmental TTP values (sdTTP) and the maximum TTP value (maxTTP) for each slice.

***Strain-Rate Cross Correlation***

SRCC was calculated using a generalized cross-correlation of strain rate time curves across each segment in the apical, mid ventricular, and basal slices. We report the maximum pairwise time-delay in milliseconds between segmental contractions in each slice as the SRCC. Perfectly synchronous contractions would have a SRCC of 0 ms, whereas dyssynchronous contractions will have longer time-delays.

***Uniformity Ratio Estimate***

We included a radial uniformity ratio estimate (RURE) in our analysis. Using a Fourier analysis, we extracted exponential power terms from segmental strain measurements in each of the apical, mid ventricular, and basal slices. As described previously, uniformity ratio estimates were calculated as,

$URE=\left( \frac{A_{0}^{2}}{A_{0}^{2}+2A_{1}^{2}} \right)^{\frac{1}{2}}$ (1)

where $A_{0}$ and $A_{1}$ are the spatial and temporal sum of the zero and first order power terms, respectively.^37^ The maximal value of RURE is 1 with all segments in the slice contracting synchronously, whereas symmetrically dyssynchronous contractions produce a value of 0.

**Comparison of Strain Techniques**

To establish a comparison between existing strain measurement techniques and deep learning synthetic strain (DLSS), patients in Necker Enfants-Malades Hospital in Paris, France were analyzed with both DLSS and commercially available feature tracking software (Medis Suite MR LV Strain; Medis Medical Imaging Systems, Leiden, Netherlands). For each patient, global radial and circumferential strain values were measured for basal, mid ventricular, and apical slices using both DLSS and feature tracking. For circumferential strain, both myocardial and endocardial measurements were investigated. Statistical analysis of this comparative analysis included the calculation of Pearson correlation coefficients, mean absolute error, and mean squared error for basal, mid ventricular, and apical slices. Results were visualized using correlation and Bland-Altman plots.

**Principal Component Analysis**

To evaluate the spatiotemporal variance within the strain data, a principal component analysis (PCA) was conducted to quantify the modes of variation between patients. PCA was conducted by performing Singular Value Decomposition of the data to a lower dimensional space. Regional strain and strain rate curves from the apical, mid ventricular, and basal slices were included, corresponding to a total of 656 data points per patient. The number of components was selected by optimizing the PCA to explain at least 75% of the total variance within the patient population.

Following decomposition, the principal modes of variation within the patient population were explored by plotting the strain curves corresponding to plus and minus two standard deviations of each mode. Furthermore, the population was partitioned using the previously identified mechanical clusters and the mean and standard deviation of PCA values for each cluster were recorded. To assess for statistically significant differences in PCA values between clusters, pairwise two-sided *t*-tests were utilized. All analysis was conducted in Python version 3.10.13 (Python Software Foundation, Wilmington, DE).

**Supplemental Results**

**Comparison of Strain Techniques**

A total of 26 patients were analyzed using both DLSS and commercial feature tracking software. DLSS and feature tracking showed a moderate to strong correlation between myocardial global circumferential strain measurements in mid ventricular (r(26)=0.47, p<0.01) and basal slices (r(26)=0.45, p<0.01). Feature tracking measurements of the endocardial layer showed even stronger correlations with DLSS, with global circumferential strain measurements in the mid ventricular (r(26)=0.53, p<0.001) and basal slices (r(26)=0.57, p<0.001) both showing strong and significant associations. Global circumferential strain measurements in the apical slices were weakly to moderately correlated (endocardial r(26)=0.20, p=0.21, myocardial r(26)=0.20, p=0.23).

Similarly, global radial strain measurements showed a moderate to strong association between DLSS and feature tracking for basal slices (r(26)=0.41, p<0.01). A weak to moderate correlation was found between DLSS and feature tracking global radial strain measurements in the mid ventricular (r(26)=0.22, p=0.17) and apical slices (r(26)=0.15, p=0.36). Correlation and Bland-Altman plots for each of these comparisons are shown in Supplemental Figures 1 and 2.

**Principal Component Analysis**

A total of 13 PCA components were necessary to explain 75% of the total variance within the patient population. To demonstrate the meaning of each PCA mode, example strain curves from the basal slice corresponding to plus and minus two standard deviations of each mode are provided in Supplemental Figure 3. PCA mode 1 corresponded to the time of peak contraction. PCA modes 2 and 3 corresponded to the strength of contraction, particularly in the lateral segments. In contrast, PCA modes 3 and 4 corresponded to dyssynchronous contractions in the anteroseptal and inferoseptal segments.

The mean and standard deviation of PCA modes per LV mechanical cluster are provided in Supplemental Table 4. Modes 1 through 5 all showed significant differences between the clusters. These findings are consistent with the primary analysis, which showed significant differences in peak strain and dyssynchrony between the LV mechanical clusters.

**Sensitivity Analysis: Progression to Pulmonary Valve Replacement**

Subsequent progression to PVR was assessed in a subset of the cohort (n=161), in which patients with a prior PVR were excluded (n=37). The mean follow-up time was 5.1±3.8 years (range 0.4-13.3 years). A total of 60 patients (37%) received a PVR during the follow-up period, with a mean time to PVR of 34.1±28.4 months (range 2–147 months) after the date of MRI examination in the original clustering analysis. Patients in cluster 1 exhibited more rapid progression to PVR than patients in cluster 3 and 4 (23±21 vs. 41±34 and 29±25 months, log-rank p<0.01), but not cluster 2 (23±21 vs 38±33, log-rank p=0.11). We show Kaplan-Meier survival curves for clusters 1, 2, 3, and 4, as well as boxplots of progression free survival time for each cluster, in Supplemental Figure 4. The results of this sensitivity analysis were consistent with the primary analysis, which also found that patients in cluster 1, which was characterized by dyssynchrony and decreased strain in the septal segments, showed a statistically significant decrease in progression free survival relative to the well-compensated patients in clusters 3 and 4.

**Supplemental Tables**

**Supplemental Table 1: MRI Short-Axis Cine Steady-State Free Precession Parameters**

| **Parameter** | **UC San Diego Health** | **Necker-Enfants**  **Malades Hospital** | **Rady Children’s Hospital** | **Inova Fairfax Hospital** | **Hospital Clínic de Barcelona** |
| --- | --- | --- | --- | --- | --- |
| Flip angle (degrees) | 55.4 (55 – 60) | 65.4 (65 – 75) | 55.2 (20 – 90) | 54.7 (45 – 55) | 45 (45 – 45) |
| Phase spatial res. (mm) | 1.8 (1.6 – 2.8) | 1.8 (1.4 – 2.1) | 2.1 (1.5 – 2.2) | 2.0 (1.6 – 2.5) | 1.8 (1.5 – 2.0) |
| Freq. spatial res. (mm) | 1.8 (1.3 – 2.4) | 1.9 (1.4 – 2.2) | 1.8 (1.2 – 2.0) | 1.9 (1.6 – 2.2) | 1.9 (1.6 – 2.5) |
| Field of view (mm) | 357 (320 – 440) | 374 (249 – 430) | 368 (280 – 450) | 354 (281 – 409) | 334 (256 – 358) |
| Slice thickness (mm) | 8.1 (8.0 – 10.0) | 8.0 (7.0 – 8.0) | 8.4 (7.0 – 10.0) | 7.9 (5.0 – 8.0) | 8.0 (8.0 – 8.0) |
| Slice gap (mm) | 1.6 (0.0 – 2.0) | 0.0 (0.0 – 0.0) | 0.1 (0.0 – 2.0) | 1.0 (0.5 – 1.0) | 0.1 (0.0 – 2.0) |
| Repetition time (msec) | 3.6 (3.3 – 4.3) | 3.4 (3.2 – 4.0) | 3.3 (2.8 – 7.1) | 3.5 (3.2 – 3.7) | 3.5 (2.9 – 3.9) |
| Echo time (msec) | 1.5 (1.2 – 1.9) | 1.5 (1.4 – 1.8) | 1.6 (1.3 – 3.4) | 1.5 (1.3 – 1.7) | 1.5 (1.1 – 1.8) |
| Echo Train Length (n) | 1 (1 – 1) | 1 (1 – 1) | 5.6 (1 – 13) | 1 (1 – 1) | 1 (1 – 1) |
| Field strength (T) | 3.0 (1.5 – 3.0) | 1.5 (1.5 – 1.5) | 1.5 (1.5 – 1.5) | 1.5 (1.5 – 3.0) | 3.0 (3.0 – 3.0) |
| Note – Data is reported as the mean, with ranges in parentheses except Field strength, which is reported as the mode, with range in parenthesis. | | | | | |

**Supplemental Table 2: Radial strain and strain rate for each cluster and the entire cohort.**

|  | **Cluster 1**  **(n = 39)** | **Cluster 2**  **(n = 29)** | | **Cluster 3**  **(n = 56)** | **Cluster 4**  **(n = 74)** | **p-value** | | **All Patients**  **(n = 198)** |
| --- | --- | --- | --- | --- | --- | --- | --- | --- |
| **Peak Radial Strain (RS)** |  |  | |  |  |  | |  |
| **Apical Slice** |  |  | |  |  |  | |  |
| Apical Septal RS (%) | 8.2 ± 15.8^3^ | 7.8 ± 6.7^34^ | | 18.4 ± 13.2^124^ | 4.5 ± 4.8^23^ | <0.001 | | 9.7 ± 12.1 |
| Apical Anterior RS (%) | 36.5 ± 14.4^2^ | 25.6 ± 9.8^134^ | | 33.7 ± 13.0^24^ | 41.2 ± 13.5^23^ | <0.001 | | 35.8 ± 14.1 |
| Apical Lateral RS (%) | 78.5 ± 25.0^24^ | 51.5 ± 17.2^134^ | | 78.4 ± 26.5^24^ | 99.3 ± 27.8^123^ | <0.001 | | 82.3 ± 30.1 |
| Apical Inferior RS (%) | 46.0 ± 20.6^4^ | 34.5 ± 13.1^34^ | | 57.5 ± 21.0^2^ | 62.5 ± 23.3^12^ | <0.001 | | 53.8 ± 23.1 |
| Apical Global RS (%) | 32.6 ± 8.1^234^ | 26.5 ± 8.2^134^ | | 40.5 ± 7.3^12^ | 42.6 ± 9.8^12^ | <0.001 | | 37.7 ± 10.4 |
|  |  |  | |  |  |  | |  |
| **Mid Ventricular Slice** |  |  | |  |  |  | |  |
| Mid Inferoseptal RS (%) | 6.5 ± 9.3^23^ | 14.1 ± 7.7^134^ | | 24.0 ± 11.2^124^ | 9.6 ± 7.5^23^ | <0.001 | | 13.7 ± 11.3 |
| Mid Anteroseptal RS (%) | 8.0 ± 7.3^34^ | 11.7 ± 10.3^3^ | | 21.4 ± 11.6^124^ | 14.2 ± 7.8^13^ | <0.001 | | 14.7 ± 10.5 |
| Mid Anterior RS (%) | 32.1 ± 10.4^23^ | 23.3 ± 6.4^14^ | | 24.9 ± 9.6^14^ | 33.1 ± 10.7^23^ | <0.001 | | 29.2 ± 10.7 |
| Mid Anterolateral RS (%) | 59.3 ± 17.0^23^ | 38.3 ± 10.3^134^ | | 47.1 ± 15.1^124^ | 62.5 ± 16.3^23^ | <0.001 | | 54.0 ± 17.8 |
| Mid Inferolateral RS (%) | 68.7 ± 19.5^2^ | 44.0 ± 11.4^134^ | | 61.9 ± 18.2^24^ | 74.1 ± 18.0^23^ | <0.001 | | 65.2 ± 20.2 |
| Mid Inferior RS (%) | 38.7 ± 15.0^34^ | 31.7 ± 9.8^34^ | | 50.2 ± 22.4^12^ | 50.0 ± 12.3^12^ | <0.001 | | 45.2 ± 17.6 |
| Mid Global RS (%) | 27.2 ± 6.2^34^ | 24.9 ± 5.4^34^ | | 33.9 ± 5.1^12^ | 35.4 ± 5.0^12^ | <0.001 | | 31.8 ± 6.8 |
|  |  |  | |  |  |  | |  |
| **Basal Slice** |  |  | |  |  |  | |  |
| Basal Inferoseptal RS (%) | 5.6 ± 6.1^234^ | 13.2 ± 6.8^13^ | | 21.5 ± 9.4^124^ | 10.2 ± 8.6^13^ | <0.001 | | 13.0 ± 10.0 |
| Basal Anteroseptal RS (%) | 10.5 ± 10.6^34^ | 15.4 ± 11.0^3^ | | 22.7 ± 11.8^12^ | 19.3 ± 12.6^1^ | <0.001 | | 18.0 ± 12.5 |
| Basal Anterior RS (%) | 35.4 ± 14.2^4^ | 34.7 ± 13.6^4^ | | 33.1 ± 14.6^4^ | 43.8 ± 14.6^123^ | <0.001 | | 37.8 ± 15.1 |
| Basal Anterolateral RS (%) | 60.7 ± 19.2^2^ | 43.8 ± 13.5^14^ | | 55.5 ± 33.9^4^ | 68.7 ± 21.1^23^ | <0.001 | | 59.8 ± 25.7 |
| Basal Inferolateral RS (%) | 69.3 ± 19.1^2^ | 47.0 ± 10.3^134^ | | 63.8 ± 18.1^24^ | 79.7 ± 25.1^23^ | <0.001 | | 68.3 ± 23.2 |
| Basal Inferior RS (%) | 42.6 ± 22.5 | 35.5 ± 8.5^34^ | | 46.9 ± 12.5^2^ | 49.2 ± 13.7^2^ | <0.001 | | 45.2 ± 15.7 |
| Basal Global RS (%) | 28.7 ± 5.4^34^ | 28.9 ± 6.7^34^ | | 35.0 ± 5.6^124^ | 37.8 ± 5.8^123^ | <0.001 | | 33.9 ± 7.0 |
|  |  |  | |  |  |  | |  |
| **Peak Radial Strain Rate (RSR)** | | |  | | | |  | |
| **Apical Slice** |  |  | |  |  |  | |  |
| Apical Septal RSR (Hz) | 1.4 ± 1.0 | 1.1 ± 0.5^3^ | | 1.5 ± 0.6^24^ | 1.2 ± 0.6^3^ | <0.01 | | 1.34 ± 0.72 |
| Apical Anterior RSR (Hz) | 1.8 ± 0.6^24^ | 1.3 ± 0.4^134^ | | 1.8 ± 0.7^24^ | 2.3 ± 0.8^123^ | <0.001 | | 1.89 ± 0.74 |
| Apical Lateral RSR (Hz) | 3.5 ± 0.8^24^ | 2.5 ± 0.7^134^ | | 3.5 ± 0.8^24^ | 4.5 ± 1.3^123^ | <0.001 | | 3.73 ± 1.21 |
| Apical Inferior RSR (Hz) | 2.2 ± 0.6^34^ | 1.9 ± 0.5^34^ | | 2.7 ± 0.6^12^ | 2.9 ± 0.7^12^ | <0.001 | | 2.55 ± 0.72 |
| Apical Global RSR (Hz) | 1.8 ± 0.4^234^ | 1.5 ± 0.4^134^ | | 2.3 ± 0.4^12^ | 2.5 ± 0.6^12^ | <0.001 | | 2.15 ± 0.60 |
|  |  |  | |  |  |  | |  |
| **Mid Ventricular Slice** |  |  | |  |  |  | |  |
| Mid Inferoseptal RSR (Hz) | 1.1 ± 0.6^3^ | 1.1 ± 0.5^3^ | | 1.8 ± 0.7^124^ | 1.2 ± 0.5^3^ | <0.001 | | 1.35 ± 0.63 |
| Mid Anteroseptal RSR (Hz) | 1.0 ± 0.5^3^ | 0.9 ± 0.4^34^ | | 1.6 ± 0.6^124^ | 1.2 ± 0.5^23^ | <0.001 | | 1.22 ± 0.59 |
| Mid Anterior RSR (Hz) | 1.6 ± 0.5^23^ | 1.1 ± 0.3^14^ | | 1.3 ± 0.4^14^ | 1.8 ± 0.6^23^ | <0.001 | | 1.52 ± 0.55 |
| Mid Anterolateral RSR (Hz) | 2.5 ± 0.5^24^ | 1.9 ± 0.5^134^ | | 2.4 ± 0.6^24^ | 3.0 ± 0.8^123^ | <0.001 | | 2.59 ± 0.75 |
| Mid Inferolateral RSR (Hz) | 3.0 ± 0.7^24^ | 2.2 ± 0.6^134^ | | 3.0 ± 0.7^24^ | 3.6 ± 0.9^123^ | <0.001 | | 3.11 ± 0.89 |
| Mid Inferior RSR (Hz) | 1.9 ± 0.5^234^ | 1.5 ± 0.4^134^ | | 2.2 ± 0.4^12^ | 2.2 ± 0.4^12^ | <0.001 | | 2.04 ± 0.50 |
| Mid Global RSR (Hz) | 1.5 ± 0.3^234^ | 1.3 ± 0.2^134^ | | 1.8 ± 0.3^12^ | 1.9 ± 0.3^12^ | <0.001 | | 1.72 ± 0.38 |
|  |  |  | |  |  |  | |  |
| **Basal Slice** |  |  | |  |  |  | |  |
| Basal Inferoseptal RSR (Hz) | 1.0 ± 0.4^3^ | 1.0 ± 0.5^3^ | | 1.7 ± 0.6^124^ | 1.2 ± 0.6^3^ | <0.001 | | 1.28 ± 0.61 |
| Basal Anteroseptal RSR (Hz) | 1.1 ± 0.5^34^ | 1.1 ± 0.5^34^ | | 1.7 ± 0.9^12^ | 1.5 ± 0.7^12^ | <0.001 | | 1.42 ± 0.76 |
| Basal Anterior RSR (Hz) | 1.6 ± 0.5^4^ | 1.5 ± 0.5^4^ | | 1.6 ± 0.6^4^ | 2.1 ± 0.5^123^ | <0.001 | | 1.78 ± 0.59 |
| Basal Anterolateral RSR (Hz) | 2.6 ± 0.6^24^ | 2.1 ± 0.7^134^ | | 2.6 ± 0.6^24^ | 3.1 ± 0.8^123^ | <0.001 | | 2.71 ± 0.79 |
| Basal Inferolateral RSR (Hz) | 3.0 ± 0.7^24^ | 2.3 ± 0.6^134^ | | 3.2 ± 0.9^24^ | 3.7 ± 0.9^123^ | <0.001 | | 3.23 ± 0.98 |
| Basal Inferior RSR (Hz) | 1.8 ± 0.4^234^ | 1.6 ± 0.2^134^ | | 2.1 ± 0.4^12^ | 2.2 ± 0.5^12^ | <0.001 | | 2.01 ± 0.47 |
| Basal Global RSR (Hz) | 1.5 ± 0.3^34^ | 1.4 ± 0.3^34^ | | 1.9 ± 0.3^124^ | 2.0 ± 0.3^123^ | <0.001 | | 1.78 ± 0.42 |
|  |  |  | |  |  |  | |  |
| Note – Data is reported as the mean and standard deviation. *P*-values are calculated using a one-way ANOVA between clusters 1, 2, 3 and 4. Significant values (*p*<0.01) are bolded. Post-hoc analysis: Significant differences (*p*<0.01) by two-sided *t*-test are indicated by superscripts (^1^=Significantly different from cluster 1; ^2^=Significantly different from cluster 2; ^3^=Significantly different from cluster 3; ^4^=Significantly different from cluster 4). RS = radial strain; RSR = radial strain rate. | | | | | | | | |

**Supplemental Table 3: Dyssynchrony metrics for each cluster and the entire cohort.**

|  | **Cluster 1**  **(n = 39)** | **Cluster 2**  **(n = 29)** | **Cluster 3**  **(n = 56)** | **Cluster 4**  **(n = 74)** | **p-value** | **All Patients**  **(n = 198)** |
| --- | --- | --- | --- | --- | --- | --- |
| **Apical Slice** |  |  |  |  |  |  |
| Apical Maximum TTP (ms) | 677 ± 137^234^ | 536 ± 258^13^ | 330 ± 113^124^ | 562 ± 200^13^ | <0.001 | 515 ± 219 |
| Apical Standard Deviation TTP (ms) | 180 ± 57^3^ | 132 ± 95^3^ | 65 ± 46^124^ | 154 ± 67^3^ | <0.001 | 131 ± 79 |
| Apical SRCC (ms) | 437 ± 136^234^ | 257 ± 197^13^ | 140 ± 110^124^ | 317 ± 150^13^ | <0.001 | 282 ± 180 |
| Apical RURE | 0.7 ± 0.1^34^ | 0.7 ± 0.1^3^ | 0.8 ± 0.1^124^ | 0.8 ± 0.1^13^ | <0.001 | 0.76 ± 0.1 |
|  |  |  |  |  |  |  |
| **Mid Ventricular Slice** |  |  |  |  |  |  |
| Mid Maximum TTP (ms) | 715 ± 124^234^ | 471 ± 170^13^ | 340 ± 97^124^ | 448 ± 192^13^ | <0.001 | 474 ± 201 |
| Mid Standard Deviation TTP (ms) | 188 ± 46^234^ | 100 ± 51^13^ | 66 ± 38^124^ | 103 ± 61^13^ | <0.001 | 109 ± 66 |
| Mid SRCC (ms) | 426 ± 152^234^ | 222 ± 188^13^ | 122 ± 93^124^ | 265 ± 160^13^ | <0.001 | 250 ± 181 |
| Mid RURE | 0.7 ± 0.1^234^ | 0.8 ± 0.1^13^ | 0.9 ± 0.1^124^ | 0.8 ± 0.1^13^ | <0.001 | 0.79 ± 0.1 |
|  |  |  |  |  |  |  |
| **Basal Slice** |  |  |  |  |  |  |
| Basal Maximum TTP (ms) | 704 ± 171^234^ | 454 ± 169^13^ | 327 ± 101^124^ | 393 ± 147^13^ | <0.001 | 445 ± 198 |
| Basal Standard Deviation TTP (ms) | 181 ± 59^234^ | 91 ± 47^13^ | 58 ± 28^124^ | 90 ± 49^13^ | <0.001 | 99 ± 63 |
| Basal SRCC (ms) | 443 ± 135^234^ | 200 ± 155^13^ | 123 ± 94^124^ | 222 ± 145^13^ | <0.001 | 234 ± 173 |
| Basal RURE | 0.7 ± 0.1^234^ | 0.8 ± 0.1^1^ | 0.9 ± 0.1^14^ | 0.8 ± 0.1^13^ | <0.001 | 0.82 ± 0.1 |
|  |  |  |  |  |  |  |
| Note – Data is reported as the mean and standard deviation. *P*-values are calculated using a one-way ANOVA between clusters 1, 2, 3, and 4. Significant values (*p*<0.01) are bolded. Post-hoc analysis: Significant differences (*p*<0.01) by two-sided *t*-test are indicated by superscripts (^1^=Significantly different from cluster 1; ^2^=Significantly different from cluster 2; ^3^=Significantly different from cluster 3; ^4^=Significantly different from cluster 4). TTP = time to peak; SRCC = radial strain rate cross correlation; RURE = radial uniformity ratio estimate. | | | | | | |

**Supplemental Table 4: Principal component analysis values per cluster.**

| **Component** | **Cluster 1** | **Cluster 2** | **Cluster 3** | **Cluster 4** | **p-value** |
| --- | --- | --- | --- | --- | --- |
| Mode 1 | -4.75±11.46^4^ | -2.76±12.02^4^ | 0.29±11.78 | 3.18±13.20^12^ | 0.017 |
| Mode 2 | -6.00±19.07^24^ | 1.91±3.45^13^ | -0.94±3.26^24^ | 2.08±3.85^13^ | **<0.001** |
| Mode 3 | -3.73±11.09^4^ | 0.00±6.19^4^ | -1.81±6.32^4^ | 2.86±6.42^123^ | **<0.001** |
| Mode 4 | -0.07±4.25^23^ | -6.25±5.03^134^ | 4.68±4.80^124^ | -0.12±4.10^23^ | **<0.001** |
| Mode 5 | -1.37±2.85^3^ | -2.95±6.43^34^ | 3.05±4.73^124^ | -0.16±5.54^23^ | **<0.001** |
| Mode 6 | 0.54±2.98 | 0.12±5.43 | 0.49±6.05 | -0.66±4.17 | 0.55 |
| Mode 7 | 1.99±3.66^34^ | 1.73±3.92^4^ | -0.02±4.48^1^ | -1.72±5.09^12^ | **<0.001** |
| Mode 8 | 1.69±4.10^4^ | 0.21±3.92 | -0.09±3.62 | -0.72±4.97^1^ | 0.10 |
| Mode 9 | 0.56±2.50 | 0.39±4.78 | 0.10±3.78 | -0.51±3.78 | 0.53 |
| Mode 10 | 0.24±3.01 | 0.80±4.20 | 0.24±3.28 | -0.72±3.63 | 0.18 |
| Note – Data is reported as the mean and standard deviation. *P*-values are calculated using a one-way ANOVA between clusters 1, 2, 3, and 4. Significant values (*p*<0.01) are bolded. Post-hoc analysis: Significant differences (*p*<0.01) by two-sided *t*-test are indicated by superscripts (^1^=Significantly different from cluster 1; ^2^=Significantly different from cluster 2; ^3^=Significantly different from cluster 3; ^4^=Significantly different from cluster 4). | | | | | |

**Supplemental Figures**

**
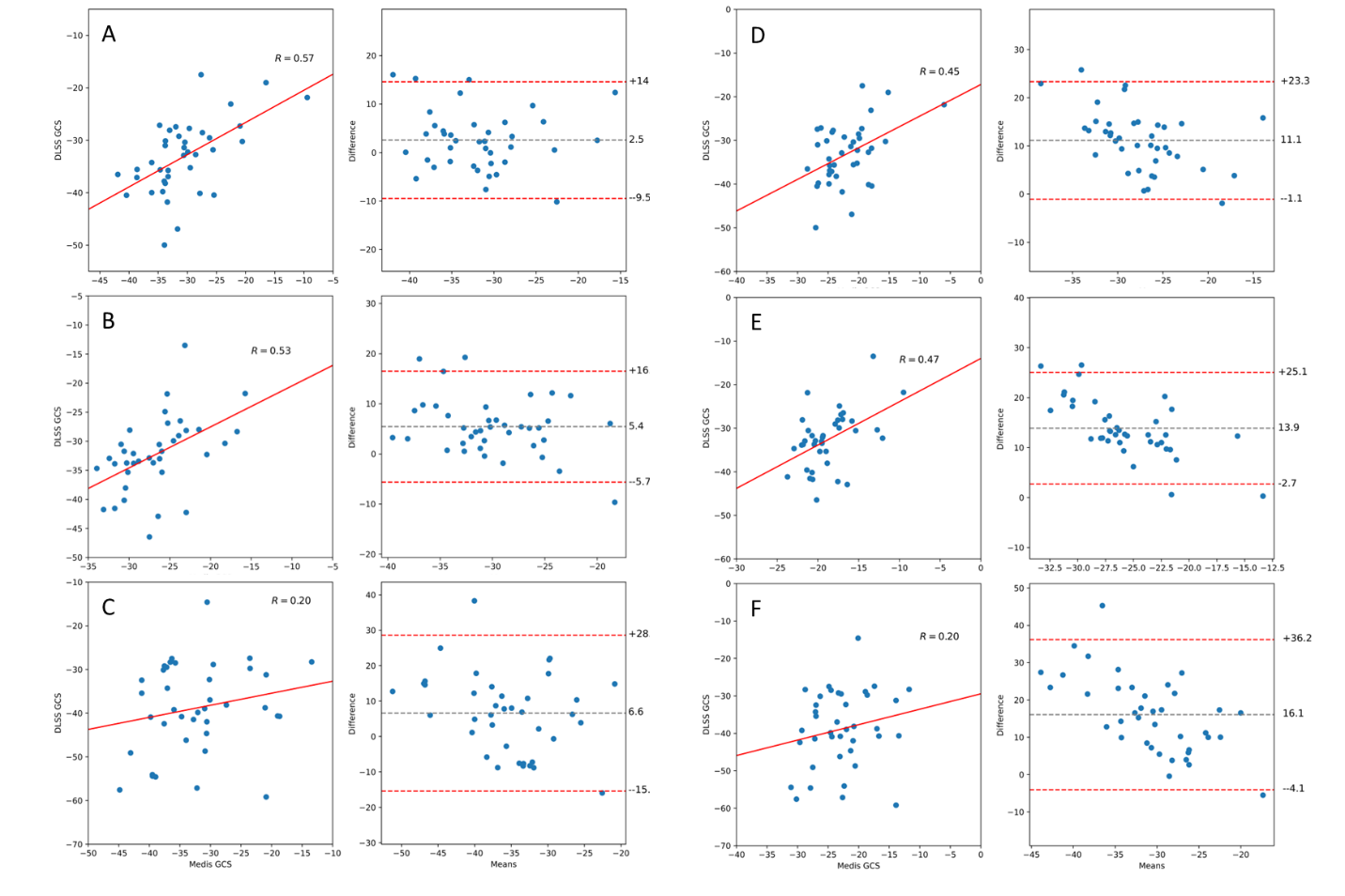
**

**Supplemental Figure 1:** Correlation and Bland-Altman plots for Deep Learning Synthetic Strain (DLSS) and feature tracking global circumferential strain measurements. Myocardial measurements from feature tracking vs. DLSS are shown for basal **(A)**, mid ventricular **(B)**, and apical slices **(C)**. Endocardial measurements from feature tracking vs. DLSS are shown for basal **(D)**, mid ventricular **(E)**, and apical slices **(F)**.

**
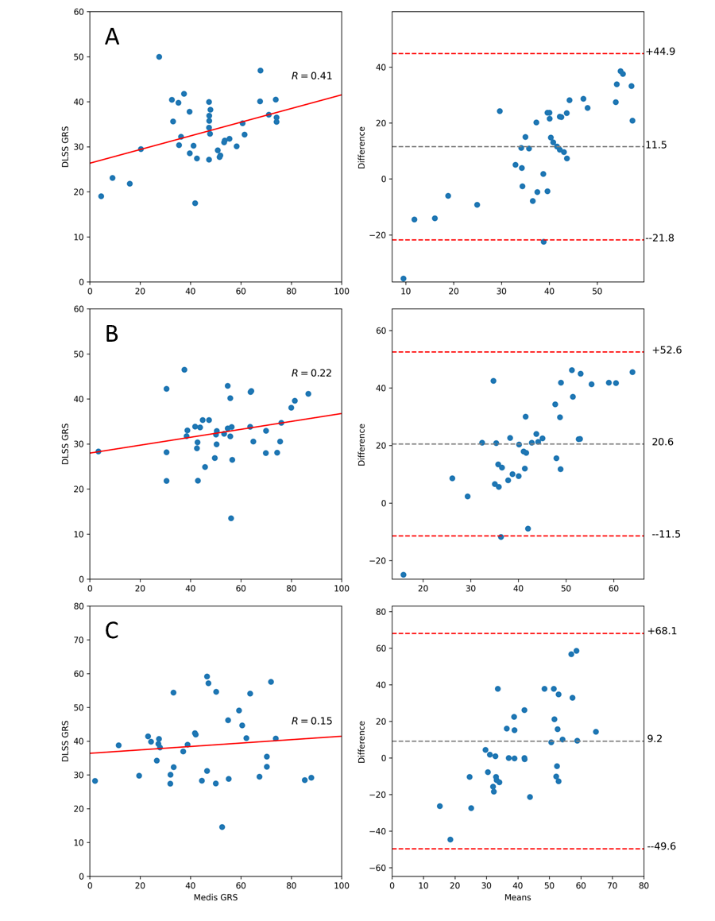
**

**Supplemental Figure 2:** Correlation and Bland-Altman plots for global radial strain measurements of the basal **(A)**, mid ventricular **(B)**, and apical slices **(C)** between Deep Learning Synthetic Strain (DLSS) and feature tracking.


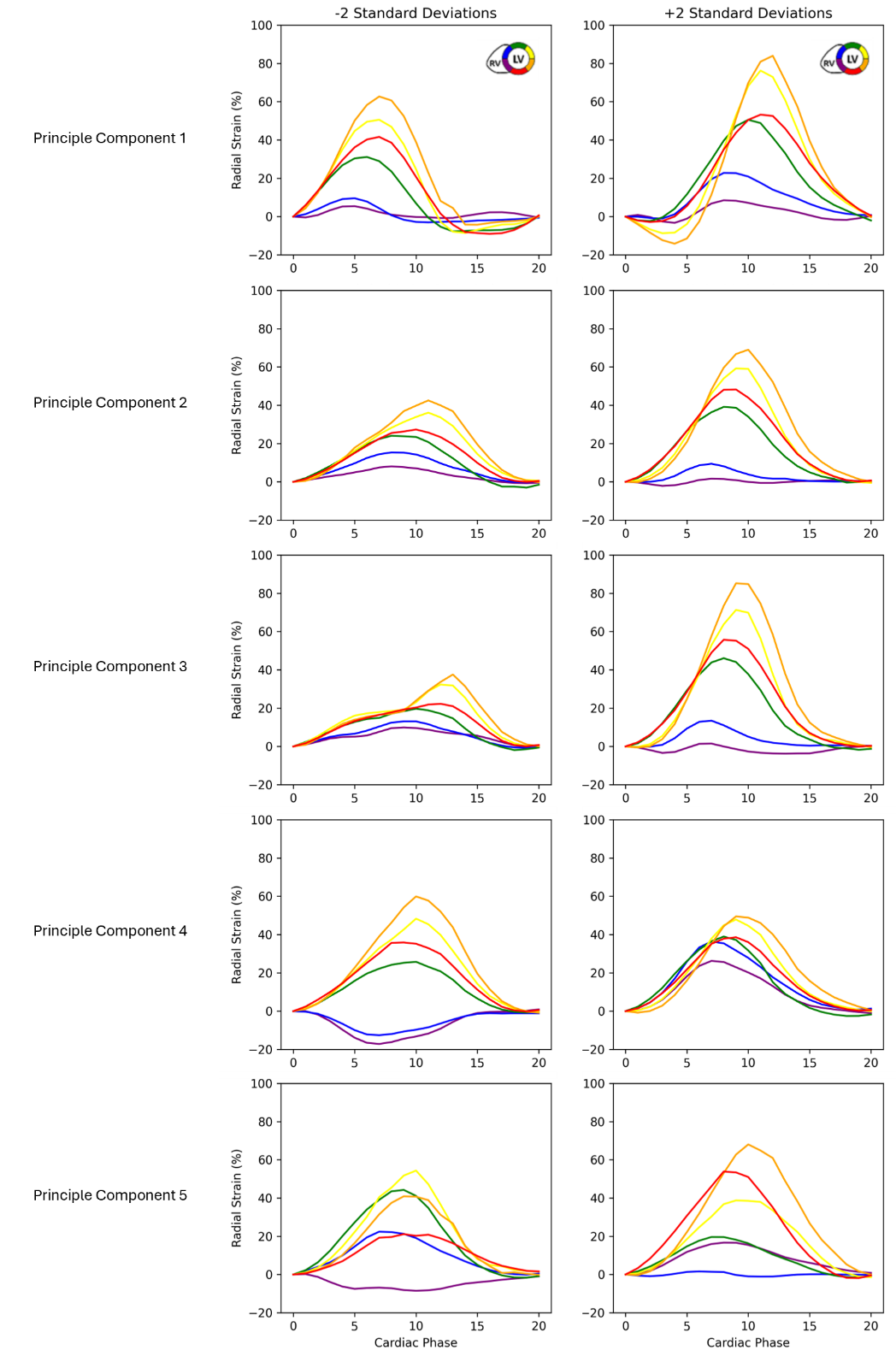


**Supplemental Figure 3:** Example strain curves corresponding to plus and minus two standard deviations for principal component analysis modes one through five.


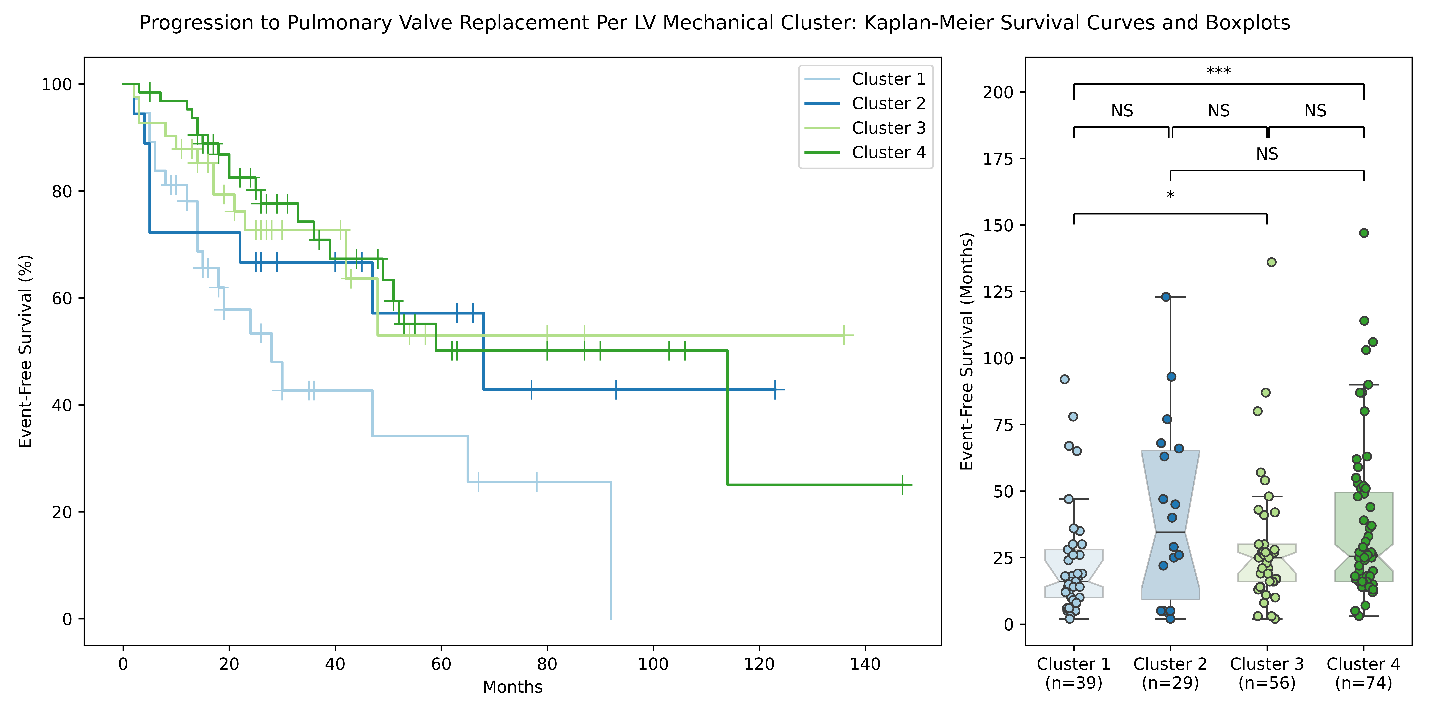


**Supplemental Figure 4:** Kaplan-Meier survival curves and boxplots showing time since MRI evaluation until progression to pulmonary valve replacement for patients in LV mechanical clusters 1, 2, 3 and 4. Patients in cluster 1, which was characterized by dyssynchrony and decreased strain in the septal segments, showed a statistically significant decrease in progression free survival relative to the well-compensated patients in cluster 3 and 4. Significance was assessed with pairwise log-rank tests. **=p<0.05; ***=p<0.001; NS=not statistically significant.*
